# Non-disclosure of HIV testing history in population-based surveys: implications for estimating a UNAIDS 90-90-90 target

**DOI:** 10.1080/16549716.2018.1553470

**Published:** 2018-12-14

**Authors:** Christopher T. Rentsch, Georges Reniers, Richard Machemba, Emma Slaymaker, Milly Marston, Alison Wringe, Jeffrey W. Eaton, Annabelle Gourlay, Brian Rice, Chodziwadziwa Whiteson Kabudula, Mark Urassa, Jim Todd, Basia Żaba

**Affiliations:** a Department of Population Health, London School of Hygiene & Tropical Medicine, London, UK; b MRC/Wits Rural Public Health and Health Transitions Research Unit (Agincourt), School of Public Health, Faculty of Health Sciences, University of the Witwatersrand, Johannesburg, South Africa; c The TAZAMA Project, National Institute for Medical Research, Mwanza, Tanzania; d Department of Infectious Disease Epidemiology, Imperial College London, London, UK; e Department of Social and Environmental Health Research, London School of Hygiene & Tropical Medicine, London, UK

**Keywords:** HIV, self-disclosure, longitudinal studies, demography, sub-Saharan Africa

## Abstract

**Background**: HIV/AIDS programmes and organisations around the world use routinely updated estimates of the UNAIDS 90-90-90 targets to track progress and prioritise further programme implementation. Any bias in these estimates has the potential to mislead organisations on where gaps exist in HIV testing and treatment programmes.

**Objective**: To measure the extent of undisclosed HIV testing history and its impact on estimating the proportion of people living with HIV (PLHIV) who know their HIV status (the ‘first 90’ of the UNAIDS 90-90-90 targets).

**Methods**: We conducted a retrospective cohort study using population-based HIV serological surveillance conducted between 2010 and 2016 and linked, directly observed HIV testing records in Kisesa, Tanzania. Generalised estimating equations logistic regression models were used to detect associations with non-disclosure of HIV testing history adjusting for demographic, behavioural, and clinical characteristics. We compared estimates of the ‘first 90’ using self-reported survey data only and augmented estimates using information from linked records to quantify the absolute and relative impact of undisclosed HIV testing history.

**Results**: Numbers of participants in each of the survey rounds ranged from 7171 to 7981 with an average HIV prevalence of 6.9%. Up to 33% of those who tested HIV-positive and 34% of those who tested HIV-negative did not disclose their HIV testing history. The proportion of PLHIV who reported knowing their status increased from 34% in 2010 to 65% in 2016. Augmented estimates including information from directly observed testing history resulted in an absolute impact of 6.7 percentage points and relative impact of 12.4%.

**Conclusions**: In this population, self-reported testing history in population-based HIV serological surveys under-estimated the percentage of HIV positives that are diagnosed by a relative factor of 12%. Research should be employed in other surveillance systems that benefit from linked data to investigate how bias may vary across settings.

## Background

The effectiveness of HIV testing and counselling (HTC) services is principally measured by the number of people living with HIV (PLHIV) who know their HIV status [1]. These services are the gateway to receiving further HIV prevention, care, and treatment services. In 2014, the Joint United Nations Programme on HIV/AIDS (UNAIDS) launched a series of targets known as ‘90-90-90’, which stated that by 2020, 90% of all PLHIV will be diagnosed (the ‘first 90’), 90% of people diagnosed with HIV will receive antiretroviral treatment (ART) (the ‘second 90’), and 90% of people receiving ART will achieve viral suppression (the ‘third 90’) [2]. Routinely updated estimates of the 90-90-90 targets assist programmes and organisations to track progress and prioritise further programme implementation.

In eastern and southern Africa, where more than half of global PLHIV [3,4] are resident, estimates of the ‘first 90’ are derived from national population-based surveys that include HIV serological testing (henceforth termed ‘sero-surveys’) [5]. When the survey includes a question directly asking respondents to report their last HIV test result, the estimate of the ‘first 90’ in the year of the survey is simply the proportion of people who reported they were diagnosed with HIV at their last HIV test out of the total number who tested HIV-positive in the survey. If the survey does not include a direct question about the knowledge of HIV status, such as Demographic and Health Surveys (DHS) [6], the estimate of the ‘first 90’ in the year of the survey is the average of two indicators: (i) the percentage of people diagnosed with HIV in the survey who report ever having been tested for HIV and receiving their last test result (upper bound), and (ii) the percentage of PLHIV on ART as reported with national ART programme data (lower bound) [5]. However, the accuracy of the upper bound estimate may be affected by respondents who are hesitant to report their HIV testing history, or by the training and ability of the interviewers to ask sensitive questions [1,7]. Thus, the current UNAIDS estimation method for the ‘first 90’ could potentially be sensitive to undisclosed HIV testing history.

In this report, we used data from a health and demographic surveillance site (HDSS) in northwest Tanzania with population-based HIV sero-surveillance and linked medical records to measure the extent of undisclosed HIV testing history among sero-survey participants who had attended a previous sero-survey or had previously registered for HIV care and treatment. We modelled associations with non-disclosure of HIV testing history and stratified results by HIV test result. Finally, we measured the discrepancy between an estimate of the ‘first 90’ using survey data and an augmented estimate using linked HIV testing history and medical records.

## Methods

### Data sources

The Kisesa observational HIV cohort study was established in 1994 and is located in a rural ward in the Magu district of Mwanza region in northwest Tanzania [8]. The study conducts annual or bi-annual rounds of household-based demographic surveillance (31 to date) and has completed 8 rounds of population-based HIV sero-surveys. During a sero-survey round, all adults aged 15 years or older living in the Kisesa HDSS coverage area are invited to attend a temporary village-based clinic for a personal interview and to give a finger-prick blood specimen to be anonymously tested for HIV. Beginning in 2007, voluntary HTC services were offered at the sero-survey clinics to those who wanted to know their HIV status, based on a second, separate blood specimen for the HIV test. Sero-survey records indicate whether the participant received their test result. Participants’ records from all sero-survey rounds are linked with a unique permanent identifier, and temporary household-based identifiers from the HDSS are also cross-referenced on each sero-survey record. This analysis included all sero-survey participants in each of the three most recent rounds: Sero 6 (2010), Sero 7 (2013), and Sero 8 (2016).

A government-run health centre is located within the Kisesa HDSS catchment area, including an HIV care and treatment centre (CTC). The CTC databases have been fully digitised, and data clerks regularly update and run data checks on these data. Medical records from the CTC have been linked to the HDSS database using point-of-contact interactive record linkage (PIRL), described elsewhere [9,10]. Ethical approvals for each of the sero-survey rounds and the PIRL study were obtained from the Tanzanian National Institute for Medical Research Lake Zone Institutional Review Board (MR/53/100/450) and the London School of Hygiene & Tropical Medicine (8852). Informed written consent was obtained from all participants.

### Estimating the ‘first 90’

The Kisesa sero-surveys, akin to DHS, include indirect questions about knowledge of HIV status by asking about HIV testing history. To determine risk factors associated with non-disclosure of HIV testing history, the regression analyses only included participants with evidence of a previous diagnostic HIV test. A previous diagnostic test was determined by the linked sero-survey records from all sero-survey rounds. We also considered patients who had registered for care at the local HIV care and treatment clinic to be diagnosed at the time of registration or as noted in their medical records. UNAIDS estimates the upper bound of the ‘first 90’ using the following two questions: (i) ‘Have you ever had HIV testing and counselling?’; and (ii) ‘Did you find out your test results after your last test?’ Those who responded affirmatively to both questions were classified as disclosing their HIV testing history. All others were classified as having an undisclosed HIV testing history.

Following UNAIDS guidelines [5], we estimated the ‘first 90’ for each sero-survey round by averaging the proportion of participants who tested HIV-positive who reported ever having been tested for HIV and receiving their last test result (upper bound), and the percentage of adult PLHIV on ART as reported with national ART programme data (lower bound). Estimates of the percentage of adult PLHIV on ART for each year coinciding with a sero-survey round were obtained from UNAIDS AIDSinfo [4]; however, these were national estimates as longitudinal, sub-national ART coverage estimates were not available. In a sensitivity analysis to account for possible misspecification of ART coverage, we determined the robustness of the estimates by increasing and decreasing the national ART coverage estimates by half, and by setting them to their theoretical maximum equal to the upper bound.

### Estimating impact

We updated the estimate of the ‘first 90’ by adding information from the linked HIV testing history and medical records. For each sero-survey round, we calculated two measures of the sensitivity of the UNAIDS estimation method to undisclosed HIV testing history:

(1) $$absolute\;impact:\;Impact_{abs}=\hat{u}-\hat{o}$$

(2) $$relative\;impact:\;impact_{rel}=(\hat{u}-\hat{o})/\hat{u}$$

where $\hat{o}$ is the original estimate of the ‘first 90’ using self-reported survey responses, and $\hat{u}$ is the updated estimate of the ‘first 90’ by augmenting $\hat{o}$ with linked data.

### Covariates

We extracted demographic, behavioural, and clinical characteristics from each sero-survey round. Demographic variables included sex, age, education level (no primary, some primary, or primary or higher), sub-village of residence (rural, peri-urban, or urban), whether the sub-village of residence has a road, and marital status (never or ever married/cohabitated). Behavioural variables included the reported number of sex partners in the last 12 months and reported condom use at last sex. Clinical variables included whether participants visited a health provider (e.g. hospital, health centre, dispensary, antenatal clinic, vaccination clinic, visit from home-based care worker, private pharmacy, or traditional healer) in the last 12 months, and for those who tested HIV-positive, whether the participant had initiated ART prior to the sero-survey as noted in CTC records, and the time since HIV diagnosis using the first HIV-positive test date in a sero-survey. For individuals who did not have a recorded positive HIV test date in a sero-survey, we used the first HIV-positive test date as listed in their medical records.

### Statistical analyses

Chi-square and Fisher’s exact tests were used to assess differences between participants with and without a previous diagnostic HIV test, as well as between participants who did and did not disclose their HIV testing history. Some participants attended more than one sero-survey round. Thus, generalised estimating equations (GEE) logistic regression models were used to account for the correlated data. We fitted crude and adjusted GEE logistic regression models for all participants to detect differences in disclosure of HIV testing history by HIV test result. We also fitted analogous models limited to participants who tested HIV-positive during the sero-survey. Statistical analyses were performed using SAS version 9.4 (SAS Institute Inc., Cary, NC, USA).

## Results

### Sample

**Figure 1.**
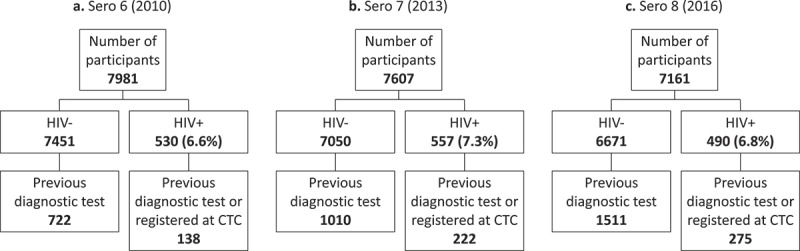
Study sample from three rounds of HIV serological surveys in Tanzania, by HIV status, 2010–2016.

There were 7981 participants (61% female) in Sero 6, 7607 participants (62% female) in Sero 7, and 7161 participants (62% female) in Sero 8, of whom 860 (10.8%), 1232 (16.2%), and 1786 (24.9%) respectively had received a diagnostic HIV test in a previous sero-survey round or registered in the CTC (Figure 1). Among participants who tested HIV-negative during a sero-survey, those who had previously received a diagnostic HIV test (i.e. repeat HIV-negative testers) were older, had more education, were from more urbanised areas, reported more sex partners in the last 12 months, reported less condom use at last sex, and reported more health service use than those who had not previously received a diagnostics HIV test (all p < 0.05) (see Supplemental Table 1). The differences between participants with and without a previous HIV diagnostic test among people who tested HIV-positive during a survey were narrower (see Supplemental Table 2), although this may be due to the smaller sample size resulting in lower statistical power. In this group, the only statistically significant difference found in multiple survey rounds was that participants who had previously received a diagnostic HIV test were from more urbanised areas than those who had not previously received a diagnostics HIV test (p < 0.04).

**Table 1.** Characteristics of survey participants with evidence of previous HIV testing, by disclosure of testing history.

	Sero 6 (2010)			Sero 7 (2013)			Sero 8 (2016)		
	Disclosed n = 679	Undisclosed n = 181	p-value	Disclosed n = 819	Undisclosed n = 413	p-value	Disclosed n = 1370	Undisclosed n = 416	p-value
HIV test result in sero									
*HIV+*	99 (72)	39 (28)	0.023	149 (67)	73 (33)	0.824	211 (77)	64 (23)	0.993
*HIV-*	580 (80)	142 (20)		670 (66)	340 (34)		1159 (77)	352 (23)	
**Demographic characteristic**									
Sex									
*Female*	407 (80)	103 (20)	0.482	470 (62)	292 (38)	<0.001	854 (74)	297 (26)	< 0.001
*Male*	270 (78)	77 (22)		349 (75)	117 (25)		516 (82)	115 (18)	
Age, years									
*15–29*	181 (79)	49 (21)	0.676	170 (64)	94 (36)	0.719	301 (72)	119 (28)	0.002
*30–49*	377 (80)	95 (20)		444 (67)	219 (33)		683 (80)	169 (20)	
*50+*	121 (77)	37 (23)		205 (67)	100 (33)		386 (75)	128 (25)	
Education level									
*No primary*	136 (70)	59 (30)	0.001	158 (51)	152 (49)	< 0.001	315 (67)	158 (33)	< 0.001
*Some primary*	88 (80)	22 (20)		97 (66)	51 (24)		156 (78)	43 (22)	
*Primary or higher*	455 (82)	100 (18)		564 (73)	210 (27)		899 (81)	215 (19)	
Sub-village of residence, type									
*Rural*	355 (75)	119 (25)	0.005	352 (59)	242 (41)	< 0.001	680 (76)	218 (24)	0.343
*Peri-urban*	184 (85)	33 (15)		234 (68)	110 (32)		358 (79)	94 (21)	
*Urban*	140 (83)	29 (17)		233 (79)	61 (21)		332 (76)	104 (24)	
Sub-village of residence, has road									
*No*	399 (75)	133 (25)	< 0.001	413 (60)	280 (40)	< 0.001	762 (76)	243 (24)	0.315
*Yes*	280 (85)	48 (15)		406 (75)	133 (25)		608 (78)	173 (22)	
Current marital status									
*Never married/cohabitated*	66 (71)	27 (29)	0.045	88 (63)	52 (37)	0.335	145 (68)	69 (32)	0.001
*Ever married/cohabitated*	613 (80)	154 (20)		731 (67)	361 (33)		1225 (78)	347 (22)	
**Behavioural characteristic**									
Number of sex partners in last 12 months									
*Don’t know/refused*	8 (44)	10 (56)	0.003	17 (46)	20 (54)	< 0.001	46 (64)	26 (36)	0.028
*0*	90 (81)	21 (19)		102 (58)	74 (42)		202 (75)	66 (25)	
*1*	462 (80)	116 (20)		599 (68)	288 (32)		1005 (77)	298 (23)	
*2 or more*	119 (78)	34 (22)		101 (77)	31 (23)		117 (82)	26 (18)	
Condom use at last sex									
*Don’t know/refused*	96 (76)	31 (24)	0.424	619 (64)	347 (36)	< 0.001	155 (69)	69 (31)	0.001
*No*	536 (79)	141 (21)		155 (72)	61 (28)		1133 (77)	336 (23)	
*Yes*	47 (84)	9 (16)		45 (90)	5 (10)		82 (88)	11 (12)	
**Clinical characteristic**									
Visited health provider in last 12 months									
*No*	84 (79)	22 (21)	0.937	105 (55)	86 (45)	< 0.002	343 (71)	142 (29)	< 0.001
*Yes*	595 (79)	159 (21)		714 (69)	327 (31)		1027 (79)	274 (21)	
**HIV+ only**									
Time since HIV diagnosis, years									
*First positive test during sero*	23 (61)	15 (39)	0.220	34 (69)	15 (31)	0.036	30 (64)	17 (36)	0.109
*<5*	48 (73)	18 (27)		62 (62)	38 (38)		79 (77)	24 (23)	
*5–9*	12 (86)	2 (14)		34 (64)	19 (36)		74 (81)	17 (19)	
*10+*	16 (80)	4 (20)		19 (95)	1 (5)		28 (82)	6 (18)	
Initiated antiretroviral therapy									
*Yes*	50 (79)	13 (21)	0.068	55 (66)	28 (34)	0.835	53 (75)	18 (25)	0.631
*No*	49 (65)	26 (35)		94 (68)	45 (32)		158 (77)	46 (23)	

Abbreviations: HIV – human immunodeficiency virus; sero – HIV serological survey

Note: all statistics are given in n(row %); differences tested for significance with chi-square (χ2) and Fisher’s exact tests.

**Table 2.** Associations with non-disclosure of HIV testing history among participants of population-based HIV serological surveys.

	All participants, n = 2747		HIV+ only, n = 454	
	cOR (95% CI)	aOR (95% CI)	cOR (95% CI)	aOR (95% CI)
HIV test result in any sero				
* HIV+ vs. HIV-*	1.10 (0.90, 1.34)	1.08 (0.87, 1.34)		
Sero round				
* Sero 8*	1.11 (0.92, 1.34)	0.86 (0.66, 1.13)	0.76 (0.48, 1.20)	0.75 (0.45, 1.24)
* Sero 7*	1.87 (1.53, 2.27)***	1.30 (0.95, 1.77)	1.22 (0.77, 1.94)	0.99 (0.55, 1.78)
* Sero 6*	1	1	1	1
HIV test result by sero round				
* HIV+ vs. HIV-, Sero 8*	1.00 (0.74, 1.35)	0.94 (0.68, 1.30)		
* HIV+ vs. HIV-, Sero 7*	0.95 (0.69, 1.29)	0.88 (0.64, 1.21)		
* HIV+ vs. HIV-, Sero 6*	1.59 (1.06, 2.40)*	1.53 (0.99, 2.36)		
**Demographic characteristic**				
Sex				
* Female*	1.46 (1.25, 1.71)***	1.54 (1.27, 1.88)***	1.38 (0.93, 2.04)	1.39 (0.87, 2.23)
* Male*	1	1	1	1
Age, years				
* 15–29*	1.25 (1.05, 1.48)*	1.18 (0.97, 1.44)	1.87 (1.22, 2.87)**	1.46 (0.92, 2.32)
* 30–49*	1	1	1	1
* 50+*	1.15 (0.96, 1.38)	1.08 (0.88, 1.32)	1.36 (0.87, 2.12)	1.43 (0.89, 2.29)
Education level				
* No primary*	2.19 (1.85, 2.58)***	2.01 (1.67, 2.41)***	1.65 (1.11, 2.44)*	1.36 (0.88, 2.10)
* Some primary*	1.23 (0.98, 1.56)	1.26 (0.99, 1.61)	1.24 (0.70, 2.19)	1.25 (0.68, 2.28)
* Primary or higher*	1	1	1	1
Sub-village of residence, type				
* Rural*	1.50 (1.24, 1.81)***	1.19 (0.89, 1.60)	1.47 (0.95, 2.27)	1.41 (0.66, 3.01)
* Peri-urban*	1.10 (0.88, 1.37)	0.94 (0.73, 1.22)	1.21 (0.74, 1.97)	1.01 (0.53, 1.92)
* Urban*	1	1	1	1
Sub-village of residence, has road				
* No*	1.51 (1.30, 1.76)***	1.35 (1.06, 1.72)*	1.27 (0.88, 1.82)	1.04 (0.55, 1.95)
* Yes*	1	1	1	1
Current marital status				
* Never married/cohabitated*	1.48 (1.20, 1.83)***	1.88 (1.43, 2.47)***	1.77 (1.00, 3.15)*	1.46 (0.74, 2.89)
* Ever married/cohabitated*	1	1	1	1
**Behavioural characteristic**				
Number of sex partners in last 12 months				
* Don’t know/refused*	2.87 (1.88, 4.36)***	1.32 (0.83, 2.08)	1.89 (0.67, 5.29)	0.99 (0.29, 3.39)
* 0*	1.48 (1.10, 1.99)*	0.82 (0.56, 1.19)	1.10 (0.55, 2.21)	0.69 (0.27, 1.77)
* 1*	1.25 (0.97, 1.59)	1.00 (0.75, 1.33)	1.08 (0.57, 2.05)	0.87 (0.39, 1.90)
* 2 or more*	1	1	1	1
Condom use at last sex				
* Don’t know/refused*	3.48 (2.29, 5.29)***	2.93 (1.84, 4.67)***	4.94 (1.50,16.31)**	4.46 (1.24,16.04)*
* No*	1.99 (1.32, 3.02)**	2.16 (1.39, 3.36)***	3.71 (1.14,12.09)*	3.19 (0.91,11.14)
* Yes*	1	1	1	1
**Clinical characteristic**				
Visited health provider in last 12 months				
* No*	1.44 (1.21, 1.70)***	1.50 (1.24, 1.80)***	1.53 (0.96, 2.45)	1.70 (1.01, 2.85)*
* Yes*	1	1	1	1
**HIV+ only**				
Times since HIV diagnosis, years				
* First positive test during sero*			3.33 (1.61, 6.87)**	3.03 (1.39, 6.62)**
* <5*			2.65 (1.37, 5.13)*	2.36 (1.18, 4.69)*
* 5–9*			1.86 (0.93, 3.75)	1.75 (0.84, 3.64)
* 10+*			1	1
Initiated antiretroviral therapy				
* Yes*			1.01 (0.70, 1.47)	1.09 (0.72, 1.64)
* No*			1	1

Abbreviations: cOR – crude unadjusted odds ratio; aOR – adjusted odds ratio; CI – confidence interval; HIV – human immunodeficiency virus; sero – HIV serological survey

*p < 0.05; **p < 0.01; ***p < 0.001

### Non-disclosure of HIV testing history

Among participants with a previous diagnostic HIV test and who tested HIV-positive in the sero-survey, 39/138 (28%) in Sero 6, 73/222 (33%) in Sero 7, and 64/275 (23%) in Sero 8 did not disclose their HIV testing history. Among participants with a previous diagnostic HIV test and who tested HIV-negative in the sero-survey, 142/722 (20%) in Sero 6, 340/1010 (34%) in Sero 7, and 352/1511 (23%) in Sero 8 did not disclose their HIV testing history. In bivariate analyses, there was a statistically significant difference between the level of non-disclosure of HIV testing history by HIV test result in Sero 6 (p = 0.02), but not in Sero 7 (p = 0.82) or Sero 8 (p = 0.99). In addition, participants with less education and who reported not knowing or who refused to report the number of sex partners in the last 12 months were more likely to not disclose their HIV testing history in all three sero-survey rounds (all p < 0.03) (Table 1).

### Regression models

After accounting for the correlated data, there was no evidence that participants who tested HIV-positive differentially disclosed their HIV testing history compared to those who tested HIV-negative during a sero-survey (crude odds ratio [cOR] 1.10, 95% confidence interval [CI] 0.90, 1.34) (Table 2). After adjusting for other covariates, non-disclosure of HIV testing history was associated with sex (females vs. males: adjusted odds ratio [aOR] 1.54, 95% CI 1.27, 1.88), education (no primary vs. primary or higher: aOR 2.01, 95% CI 1.67, 2.41), marital status (never vs. ever married/cohabitated: aOR 1.88, 95% CI 1.43, 2.47), and reported condom use at last sex (do not know/refused to answer vs. yes: aOR 2.93, 95% CI 1.84, 4.67; no vs. yes: aOR 2.16, 95% CI 1.39, 3.36). There were no significant associations between non-disclosure of HIV testing history and age, sub-village of residence, and reported number of sex partners in the last 12 months, after adjusting for other covariates.

Among participants who tested HIV-positive during the sero-survey, participants’ whose positive test during the survey was their first had 3 times more odds (aOR 3.03, 95% CI 1.39, 6.62) and those with a first recorded diagnosis of less than 5 years had 2.4 times more odds (aOR 2.36, 95% CI 1.18, 4.69) to not disclose their HIV testing history than their counterparts who had first been diagnosed over 10 years prior to the survey. There was no evidence of an association between non-disclosure of HIV testing history and whether the participant was on ART (aOR 1.08, 95% CI 0.72, 1.62).

### Implications for the estimate of the ‘first 90’

**Figure 2.**
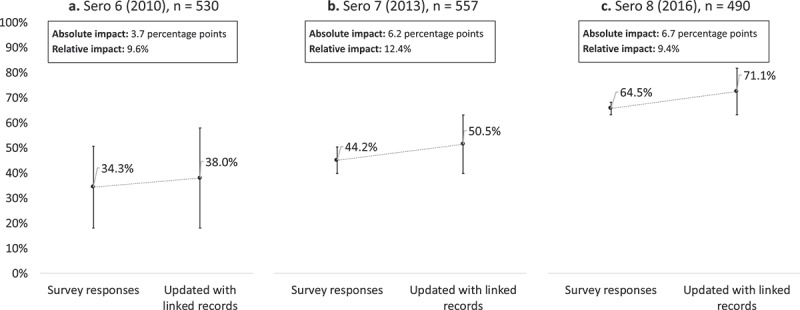
Impact of non-disclosure of HIV testing history on the proportion of PLHIV who know their status (the ‘first 90’ of the UNAIDS 90-90-90 target) using population-based HIV serological survey data and linked clinic data in Kisesa, Tanzania, 2010–2016.

Note: the ‘first 90’ for each sero-survey round was estimated by averaging the proportion of participants who tested HIV-positive and reported ever having been tested for HIV and receiving their last test result (upper bound), and the percentage of adult PLHIV on ART as reported with national ART programme data (lower bound). The bars presented in this figure represent those bounds and are not estimations of error around the ‘first 90’.

The number of participants who tested HIV-positive was 530 (6.6%) in Sero 6, 557 (7.3%) in Sero 7, and 490 (6.8%) in Sero 8. Of these, the number of participants who reported having ever been tested for HIV and received their last HIV test result (the upper bound of the ‘first 90’ estimate) was 268 (50.6%) in Sero 6, 275 (49.4%) in Sero 7, and 328 (66.9%) in Sero 8. Estimates of ART coverage (the lower bound of the ‘first 90’ estimate) in Tanzania for each year coinciding with each sero-survey round were 18% in 2010, 39% in 2013, and 62% in 2016. Thus, cross-sectional estimates of the ‘first 90’ using self-reported survey data combined with the national ART estimates were 34.3% in 2010, 44.2% in 2013, and 64.5% in 2016 (Figure 2). However, there was evidence from the linked records that some patients who tested positive during the sero-survey did not disclose their HIV testing history (39 participants in Sero 6, 70 participants in Sero 7, and 65 participants in Sero 8). After augmenting the estimates of the upper bound with this information, the estimate of the ‘first 90’ increased to 38.0% in 2010, 50.5% in 2013, and 71.1% in 2016. These increases corresponded to an absolute impact in the ‘first 90’ of 3.7 percentage points in Sero 6, 6.2 percentage points in Sero 7, and 6.7 percentage points in Sero 8, and a relative impact of 9.6% in Sero 6, 12.4% in Sero 7, and 9.4% in Sero 8.

In sensitivity analyses, decreasing estimates of ART coverage by half resulted in increased estimates of relative impact to 10.9% in Sero 6, 15.4% in Sero 7, and 12.0% in Sero 8; increasing estimates of ART coverage by 50% resulted in decreased estimates relative impact to 8.6% in Sero 6, 10.4% in Sero 7, and 7.7% in Sero 8; and setting the lower bound equal to the upper bound resulted in the highest estimates of relative impact to 12.6% in Sero 6, 20.2% in Sero 7, and 16.6% in Sero 8.

## Discussion

Due to non-disclosure of HIV testing history, self-reported HIV testing history under-estimated the ‘first 90’ of the widely used UNAIDS 90-90-90 targets by 9.4% to 12.4% between 2010 and 2016 in a rural Tanzanian community. The size of the relative impact was directly related to the proportion of participants who did not disclose their HIV testing history – more non-disclosures resulted in higher impact. In addition, our estimates of the number of individuals who received a previous HIV diagnostic test or HIV care are likely to be under-estimated since we did not capture HIV testing that occurs outside the sero-surveys or HIV care outside the Kisesa HDSS area. Because self-reports of HIV diagnosis factor into the current UNAIDS estimation of the ‘first 90’ they are likely to be under-estimated, which may ultimately affect our understanding of the gaps in HIV care and treatment programmes and misallocate resources. Other estimation methods that utilise self-reported HIV testing history may be similarly affected.

The upper bound of the UNAIDS estimate of the ‘first 90’ (proportion reporting ever tested and receiving last test result) includes individuals who may truly not know their HIV-positive status because the last test result they received was negative (i.e. sero-converters), which is why UNAIDS uses this as an upper bound rather than a direct estimate of knowledge of HIV status. The rationale for using ART coverage as the lower bound is that the proportion of PLHIV who are diagnosed (‘first 90’) cannot be lower than the proportion of PLHIV receiving ART. We found that changes in ART coverage drove the increase in the ‘first 90’ over the study period as it increased from 18% to 62% (a 224% relative change) compared to an increase from 58% to 80% (a 39% relative change) in the upper bound. While estimates of ART coverage were not available at the sub-national level, we accounted for potential measurement errors in sensitivity analyses and found that setting ART coverage equal to the upper bound increased the estimate of relative impact in the ‘first 90’ to 12.6–20.2% over the study period.

The proportion of PLHIV who reported knowing their HIV sero-status doubled from 34% in 2010 to 65% in 2016 in this rural Tanzanian community. There is a lack of longitudinal, national or sub-national estimates of the ‘first 90’ or its components available to which we can compare our findings. UNAIDS has estimated that 70% of PLHIV in Tanzania knew their sero-status in 2016 [3,4], which is similar to our sub-national estimate in that year but cannot be directly compared. A national survey conducted by the National Bureau of Statistics in 2011 showed that 54.1% of those surveyed in the Mwanza region had ever been tested for HIV and received their last test result [11], which is reasonably consistent with our finding of 50.6% of participants surveyed in the 2010 survey.

There were substantial levels of non-disclosure of HIV testing history in this sample. Between 1 in 3 and 1 in 5 participants did not accurately report their HIV testing history during a sero-survey. Interestingly, we did not find any evidence that participants who tested HIV-positive were more or less likely to disclose their HIV testing history than those who tested HIV-negative after adjusting for other factors. In unadjusted analyses, not knowing or refusing to indicate the number of sex partners in the last 12 months or condom use at last sex had the highest associations with non-disclosure. In the adjusted model, not knowing or refusing to indicate whether a condom was used at last sex remained independently associated with non-disclosure. Being prepared to answer sensitive questions about sexual behaviour may indicate a person’s willingness to be open about other sensitive topics, such as HIV testing history. Those who reported no condom use at last sex were more likely to be female, which was also associated with non-disclosure of HIV testing history. Several studies have shown women perceive and are targets of HIV-related stigma more than men [12–15], which may impede their willingness to discuss their testing history. Similarly, it is plausible that some participants prefer to deny that they have had an HIV test because the act of testing – even if HIV-negative – may suggest perceived risk and thus certain risk behaviours. In addition, the women in our sample were significantly younger and reported less education than men, both factors which were associated with non-disclosure of HIV testing history. Even though women had higher participation rates in the Kisesa sero-surveys than men, we hypothesise that men who self-select to participate may be more comfortable discussing HIV testing history, and more broadly that participation may not correlate with disclosure equally among men and women. Finally, there is a potential for recall bias among all participants since sero-survey rounds are typically three years apart.

In a model limited to participants who tested HIV-positive during a sero-survey, those who were first diagnosed with HIV infection during the sero-survey and those with a recent diagnosis of less than 5 years were up to 3 times more likely to not disclose their HIV infection compared to their counterparts who had lived with HIV for at least 10 years prior to the survey. There is some evidence among PLHIV in Tanzania that time since HIV diagnosis is negatively correlated with internalised stigma [16], and that HIV-related stigma is significantly associated with concealment of HIV status [16,17]. Therefore, individuals who have been aware of their positive HIV status for longer durations of time may have come to terms with their HIV status and were more comfortable to report their HIV testing history.

Our study had limitations. First, we were not able to take into account the specificity of self-reporting. However, if individuals reported testing when in fact they have not, this would decrease the impact of non-disclosure of testing history on the UNAIDS estimation method. Further research identifying and measuring the impact of false positives on the estimate of the ‘first 90’ will become possible as linkages between testing facilities and sero-surveys continue to expand within and around the Kisesa surveillance area. Second, we had a relatively small sample size of PLHIV and therefore lacked sufficient power to detect significant associations with non-disclosure of HIV testing history among PLHIV. Third, participation rates in the sero-surveys have declined over time to 43% of eligible adults residing in the surveillance population during the most recent survey in 2016. However, all participants agreed to provide a blood spot for anonymous HIV testing and 99% further opted to receive their results. Individuals who choose to participate may be differentially inclined to report HIV testing history than those who do not participate. Comparative research should be employed in other HDSS sites to investigate how the corrective factor in the measurement of the ‘first 90’ may vary between settings.

## Conclusions

There was substantial non-disclosure of previous HIV testing history in population-based surveys from Tanzania, which resulted in an under-estimate of the first UNAIDS 90-90-90 target between 9.4% and 12.4%. There were likely previous HIV diagnostic tests not captured in this analysis, and therefore the impact estimates are likely to still be under-estimated. The factor most associated with non-disclosure of HIV testing history was refusing to answer other sensitive questions, a finding that could potentially be used to augment estimates of the ‘first 90’ derived from other population-based surveys that do not benefit from linked HIV testing and medical records. Comparative research should be employed in other HDSS sites that benefit from linked HIV testing and clinical data to investigate how the corrective factor may vary between settings.

## Paper context

Any bias in estimating the UNAIDS 90-90-90 targets has the potential to mislead organisations on where gaps exist in HIV testing and treatment programmes. We show that current guidelines under-estimated the percentage diagnosed in a population by a relative factor of 12% and may be greater. This paper highlights the need for updated algorithms to better estimate a widely used target that assists organisations to track progress and prioritise further programme implementation.
