# Supplementary material for: Non-disclosure of HIV testing history in population-based surveys: implications for estimating a UNAIDS 90-90-90 target
Source: Glob Health Action. 2018 Dec 14;11(1):1553470. doi: 10.1080/16549716.2018.1553470 (PMC6300092; doi:10.1080/16549716.2018.1553470)
Supplement: Supplemental Material [file ZGHA_A_1553470_SM0418.zip › ST1_v1.docx]

| **Supplemental Table 1.** Characteristics among survey participants tested HIV-negative, by whether they had a previous diagnostic HIV test | | | | | | | | | | | |
| --- | --- | --- | --- | --- | --- | --- | --- | --- | --- | --- | --- |
|  | **Sero 6 (2010)** | | |  | **Sero 7 (2013)** | | |  | **Sero 8 (2016)** | | |
|  | **No previous test** | **Previous test** |  |  | **No previous test** | **Previous test** |  |  | **No previous test** | **Previous test** |  |
|  | **n=6,729** | **n=722** | **p-value** |  | **n=6,040** | **n=1,010** | **p-value** |  | **n=5,160** | **n=1,511** | **p-value** |
| **Demographic characteristic** |  |  |  |  |  |  |  |  |  |  |  |
| Sex |  |  |  |  |  |  |  |  |  |  |  |
| *Female* | 4,075 (61) | 413 (57) | 0.0544 |  | 3,682 (61) | 598 (59) | 0.2730 |  | 3,138 (61) | 956 (63) | 0.1023 |
| *Male* | 2,609 (39) | 308 (43) |  |  | 2,328 (39) | 408 (41) |  |  | 1,997 (39) | 551 (37) |  |
| Age, years |  |  |  |  |  |  |  |  |  |  |  |
| *15-29* | 3,743 (56) | 200 (28) | <0.0001 |  | 3,284 (54) | 216 (21) | <0.0001 |  | 2,845 (55) | 385 (25) | <0.0001 |
| *30-49* | 1,743 (26) | 389 (54) |  |  | 1,551 (26) | 535 (53) |  |  | 1,248 (24) | 696 (46) |  |
| *50+* | 1,243 (18) | 133 (18) |  |  | 1,205 (20) | 259 (26) |  |  | 1,067 (21) | 430 (28) |  |
| Education level |  |  |  |  |  |  |  |  |  |  |  |
| *No primary* | 1,955 (29) | 147 (20) | <0.0001 |  | 1,752 (29) | 236 (23) | 0.0002 |  | 1,380 (27) | 365 (24) | 0.1285 |
| *Some primary* | 1,109 (16) | 93 (13) |  |  | 786 (13) | 122 (12) |  |  | 571 (11) | 170 (11) |  |
| *Primary or higher* | 3,665 (54) | 482 (67) |  |  | 3,502 (58) | 652 (65) |  |  | 3,209 (62) | 976 (65) |  |
| Sub-village of residence, type |  |  |  |  |  |  |  |  |  |  |  |
| *Rural* | 3,885 (58) | 418 (58) | 0.0352 |  | 3,877 (64) | 493 (49) | <0.0001 |  | 2,817 (55) | 767 (51) | 0.0030 |
| *Peri-urban* | 1,415 (21) | 175 (24) |  |  | 1,136 (19) | 276 (27) |  |  | 1,092 (21) | 380 (25) |  |
| *Urban* | 1,429 (21) | 129 (18) |  |  | 1,027 (17) | 241 (24) |  |  | 1,251 (24) | 364 (24) |  |
| Sub-village of residence, has road | |  |  |  |  |  |  |  |  |  |  |
| *No* | 4,277 (64) | 457 (63) | 0.8885 |  | 4,187 (69) | 562 (56) | <0.0001 |  | 3,062 (59) | 849 (56) | 0.0286 |
| *Yes* | 2,452 (36) | 265 (37) |  |  | 1,853 (31) | 448 (44) |  |  | 2,098 (41) | 662 (44) |  |
| Current marital status |  |  |  |  |  |  |  |  |  |  |  |
| *Never married/cohabitated* | 2,453 (36) | 86 (12) | <0.0001 |  | 2,272 (38) | 120 (12) | <0.0001 |  | 2,067 (40) | 192 (13) | <0.0001 |
| *Ever married/cohabitated* | 4,276 (64) | 636 (88) |  |  | 3,768 (62) | 890 (88) |  |  | 3,093 (60) | 1,319 (87) |  |
|  |  |  |  |  |  |  |  |  |  |  |  |
| **Behavioural characteristic** |  |  |  |  |  |  |  |  |  |  |  |
| Number of sex partners in last 12 months | |  |  |  |  |  |  |  |  |  |  |
| *Don't know/refused* | 1,446 (21) | 14 (2) | <0.0001 |  | 1,382 (23) | 31 (3) | <0.0001 |  | 1,342 (26) | 62 (4) | <0.0001 |
| *0* | 1,006 (15) | 82 (11) |  |  | 926 (15) | 122 (12) |  |  | 661 (13) | 190 (13) |  |
| *1* | 3,741 (56) | 489 (68) |  |  | 3,343 (55) | 741 (73) |  |  | 2,880 (56) | 1,135 (75) |  |
| *2 or more* | 536 (8) | 137 (19) |  |  | 389 (6) | 116 (11) |  |  | 277 (5) | 124 (8) |  |
| Condom use at last sex |  |  |  |  |  |  |  |  |  |  |  |
| *Don't know* | 2,454 (36) | 95 (13) | <0.0001 |  | 4,852 (80) | 792 (78) | 0.2158 |  | 1,765 (34) | 167 (11) | <0.0001 |
| *No* | 3,910 (58) | 581 (80) |  |  | 987 (16) | 175 (17) |  |  | 3,162 (61) | 1,270 (84) |  |
| *Yes* | 365 (5) | 46 (6) |  |  | 201 (3) | 43 (4) |  |  | 233 (5) | 74 (5) |  |
|  |  |  |  |  |  |  |  |  |  |  |  |
| **Clinical characteristic** |  |  |  |  |  |  |  |  |  |  |  |
| Visited health provider in last 12 months | |  |  |  |  |  |  |  |  |  |  |
| *No* | 1,102 (16) | 96 (13) | 0.0322 |  | 1,226 (20) | 166 (16) | 0.0043 |  | 1,618 (31) | 422 (28) | 0.0110 |
| *Yes* | 5,627 (84) | 626 (87) |  |  | 4,814 (80) | 844 (84) |  |  | 3,541 (69) | 1,089 (72) |  |
| Abbreviations: HIV - human immunodeficiency virus; sero - HIV serological survey | | | | | | | | | | | |
| Note: all statistics are given in n(row %); differences tested for significance with chi-square (χ2) and Fisher's exact tests | | | | | | | | | | | |
