# Supplementary material for: Non-disclosure of HIV testing history in population-based surveys: implications for estimating a UNAIDS 90-90-90 target
Source: Glob Health Action. 2018 Dec 14;11(1):1553470. doi: 10.1080/16549716.2018.1553470 (PMC6300092; doi:10.1080/16549716.2018.1553470)
Supplement: Supplemental Material [file ZGHA_A_1553470_SM0418.zip › ST2_v1.docx]

| **Supplemental Table 2.** Characteristics among survey participants tested HIV-positive, by whether they had a previous diagnostic HIV test | | | | | | | | | | | |
| --- | --- | --- | --- | --- | --- | --- | --- | --- | --- | --- | --- |
|  | **Sero 6 (2010)** | | |  | **Sero 7 (2013)** | | |  | **Sero 8 (2016)** | | |
|  | **No previous test** | **Previous test** |  |  | **No previous test** | **Previous test** |  |  | **No previous test** | **Previous test** |  |
|  | **n=392** | **n=138** | **p-value** |  | **n=335** | **n=222** | **p-value** |  | **n=215** | **n=275** | **p-value** |
| **Demographic characteristic** | |  |  |  |  |  |  |  |  |  |  |
| Sex |  |  |  |  |  |  |  |  |  |  |  |
| *Female* | 269 (69) | 97 (71) | 0.5562 |  | 219 (66) | 164 (74) | 0.0483 |  | 147 (69) | 195 (71) | 0.5957 |
| *Male* | 123 (31) | 39 (29) |  |  | 113 (34) | 58 (26) |  |  | 67 (31) | 80 (29) |  |
| Age, years |  |  |  |  |  |  |  |  |  |  |  |
| *15-29* | 120 (31) | 30 (22) | 0.1177 |  | 87 (26) | 48 (22) | 0.4379 |  | 49 (23) | 35 (13) | 0.0012 |
| *30-49* | 216 (55) | 83 (60) |  |  | 188 (56) | 128 (58) |  |  | 125 (58) | 156 (57) |  |
| *50+* | 56 (14) | 25 (18) |  |  | 60 (18) | 46 (21) |  |  | 41 (19) | 84 (31) |  |
| Education level |  |  |  |  |  |  |  |  |  |  |  |
| *No primary* | 135 (34) | 48 (35) | 0.5940 |  | 130 (39) | 74 (33) | 0.4197 |  | 92 (43) | 108 (39) | 0.6587 |
| *Some primary* | 62 (16) | 17 (12) |  |  | 37 (11) | 26 (12) |  |  | 24 (11) | 29 (11) |  |
| *Primary or higher* | 195 (50) | 73 (53) |  |  | 168 (50) | 122 (55) |  |  | 99 (46) | 138 (50) |  |
| Sub-village of residence, type | |  |  |  |  |  |  |  |  |  |  |
| *Rural* | 208 (53) | 56 (41) | 0.0382 |  | 194 (58) | 101 (46) | 0.0049 |  | 111 (52) | 131 (48) | 0.3348 |
| *Peri-urban* | 99 (25) | 42 (30) |  |  | 66 (20) | 68 (31) |  |  | 44 (20) | 72 (26) |  |
| *Urban* | 85 (22) | 40 (29) |  |  | 75 (22) | 53 (24) |  |  | 60 (28) | 72 (26) |  |
| Sub-village of residence, has road | |  |  |  |  |  |  |  |  |  |  |
| *No* | 237 (60) | 75 (54) | 0.2096 |  | 221 (66) | 131 (59) | 0.0953 |  | 123 (57) | 156 (57) | 0.9148 |
| *Yes* | 155 (40) | 63 (46) |  |  | 114 (34) | 91 (41) |  |  | 92 (43) | 119 (43) |  |
| Current marital status |  |  |  |  |  |  |  |  |  |  |  |
| *Never married/cohabitated* | 34 (9) | 7 (5) | 0.1733 |  | 41 (12) | 20 (9) | 0.2321 |  | 23 (11) | 22 (8) | 0.3048 |
| *Ever married/cohabitated* | 358 (91) | 131 (95) |  |  | 294 (88) | 202 (91) |  |  | 192 (89) | 253 (92) |  |
|  |  |  |  |  |  |  |  |  |  |  |  |
| **Behavioural characteristic** | |  |  |  |  |  |  |  |  |  |  |
| Number of sex partners in last 12 months | | |  |  |  |  |  |  |  |  |  |
| *Don't know/refused* | 15 (4) | 4 (3) | 0.5641 |  | 7 (2) | 6 (3) | 0.2332 |  | 7 (3) | 10 (4) | 0.3461 |
| *0* | 63 (16) | 29 (21) |  |  | 59 (18) | 54 (24) |  |  | 47 (22) | 78 (28) |  |
| *1* | 261 (67) | 89 (64) |  |  | 239 (71) | 146 (66) |  |  | 148 (69) | 168 (61) |  |
| *2 or more* | 53 (14) | 16 (12) |  |  | 30 (9) | 16 (7) |  |  | 13 (6) | 19 (7) |  |
| Condom use at last sex |  |  |  |  |  |  |  |  |  |  |  |
| *Don't know* | 78 (20) | 32 (23) | 0.6910 |  | 264 (79) | 174 (78) | 0.9899 |  | 39 (18) | 57 (21) | 0.6070 |
| *No* | 287 (73) | 96 (70) |  |  | 61 (18) | 41 (18) |  |  | 164 (76) | 199 (72) |  |
| *Yes* | 27 (7) | 10 (7) |  |  | 10 (3) | 7 (3) |  |  | 12 (6) | 19 (7) |  |
|  |  |  |  |  |  |  |  |  |  |  |  |
| **Clinical characteristic** |  |  |  |  |  |  |  |  |  |  |  |
| Visited health provider in last 12 months | | |  |  |  |  |  |  |  |  |  |
| *No* | 49 (13) | 10 (7) | 0.0915 |  | 49 (15) | 25 (11) | 0.2519 |  | 59 (27) | 63 (23) | 0.2495 |
| *Yes* | 343 (88) | 128 (93) |  |  | 286 (85) | 197 (89) |  |  | 156 (73) | 212 (77) |  |
| Abbreviations: HIV - human immunodeficiency virus; sero - HIV serological survey | | | | | | | | | | | |
| Note: all statistics are given in n(row %); differences tested for significance with chi-square (χ2) and Fisher's exact tests | | | | | | | | | | | |
